# Patient Experience Return on Investment in Ophthalmology Services

**Published:** 2019

**Authors:** Mădălina Gheorghe Consuela

**Affiliations:** * Philologist, Authorized translator

“Return on Investment” (ROI) is a performance measure used to evaluate the efficiency of an investment or compare the efficiency of a number of different investments. To calculate ROI, the benefit (or return) of an investment is divided by the cost of the investment. 

The result is expressed as a percentage or a ratio [**1**]. Briefly, ROI is what we get for what we pay. 

In health care services, ROI is a measure for better patient experience that, implicitly, leads to a higher patient satisfaction. A higher patient satisfaction is shaped by compassion, empathy, concern, and, respect. 

In ophthalmology services, some organizations are known for investing heavily in patient experience, especially in building a relationship-centered communication between physicians and patients, as well as, by becoming “servicescape” [**2**] oriented towards using experiential marketing strategies. This philosophy seems to make not only ethical but also financial sense, because communication and empathy may bring fourth, positive emotions in consumers of ophthalmology services. Moreover, consumer oriented services may also be shaped by specific organizational factors such as safety, quality, image, and reputation, which may have as outcome several competitive advantages.

Consequently, as the health care competitive environment increases, experts are looking for new and effective methods of engaging consumers by using experiential marketing strategies and, at the same time, make them loyal to the organization or the service provider [**3**]. Besides building loyalty, a core outcome of the engagement process remains the industry’s shift towards value-based care. 

A value-based consumer care orientation has revealed ROI implications linked to increased patient loyalty, reduced malpractice claims, improved efficiency and increased physician satisfaction. 

At the same time, the link between culture-driven internal satisfaction (among staff and providers) and established positive financial outcomes, may show the implications on how Integrated Loyalty Systems’ (ILS) Patient Experience Improvement Process fits into the value creation chain. ILS targets the first links in the value creation chain, helping in shaping and implementing an organizational culture that would create positive experiences for employees and providers, so that, in their turn, they may deliver positive, consumer memorable experiences that impact profitability, and becomes visible in the ROI. 

With profitability and organizational “success” directly connected to patient satisfaction, it is easy to see how the impact of a sustained cultural improvement effort will have a positive influence on an organization’s profitability. The collateral improvements resulting from the enhanced culture combined with a positive experiential consumer strategy and a value oriented service, reflects a worthwhile return on the investment [**4**]. 

**Assist. Prof. Gheorghe Consuela-Mădălina, PhD,** **Philologist, Authorized translator**
